# The Associations Between Income and Depression: A Cross‐National Analysis

**DOI:** 10.1155/da/5511191

**Published:** 2026-08-21

**Authors:** Rachel Li, Xiantong Yang

**Affiliations:** ^1^ Department of Psychology, University of Washington, Seattle, Washington, USA, washington.edu; ^2^ School of Psychology, Fujian Normal University, Fuzhou, Fujian, China, fjnu.edu.cn; ^3^ Department of Mathematics, Science, and Social Studies Education, University of Georgia, Athens, Georgia, USA, uga.edu

**Keywords:** cross-national study, depression, income, loneliness, mediation analysis, network analysis

## Abstract

Depression remains one of the most prevalent global mental health challenges, while prior research has often examined its determinants through fragmented and isolated perspectives. A multidimensional understanding of how biological, psychological, social, and sociocultural factors jointly shape depression risk remains limited, particularly across national contexts. Using data from three nationally representative cohorts in the United Kingdom (Understanding Society: The UK Household Longitudinal Study), the United States (Midlife in the United States), and China (China Health and Retirement Longitudinal Study), we applied network analysis and parallel mediation analysis to examine the relative importance of multidimensional factors associated with depression and to assess pathways associated with income and depressive symptoms. Across all cohorts, psychological factors showed the strongest associations with depression. Lower income was directly related to higher depression and indirectly related through parallel pathways involving loneliness and disability. Loneliness emerged as the most consistent mediator across cohorts. These findings provide an integrated view of depression risk and highlight the potential role of psychological mechanisms in linking socioeconomic disadvantage to mental health vulnerability across populations.

## 1. Introduction

Depression is one of the most pressing global mental health problems and remains a leading cause of disability worldwide, impacting people’s functioning and quality of life [1, 2]. Global estimates indicate that depressive disorder impacts ~5.7% of adults around the world [2]. As a result, global and national health authorities have identified depression as a major contributor to disease burden [3, 4]. Although numerous medical and psychosocial treatments are available these days, the global prevalence associated with depression has not shown a sustained decline. This pattern suggests that current strategies may not fully address the multifactorial origins of depression [1, 5]. This limitation is reflected by the absence of clear evidence regarding how different categories of risk factors are organized, weighted, and distributed in relation to depression at the population level.

Accordingly, the present study addresses three unresolved questions: how major categories of risk factors for depression can be organized within a multidimensional framework, which dimensions contribute most strongly to depression at the population level, and whether the relative importance of these dimensions is consistent across different sociocultural contexts. In particular, although a wide range of biological, psychological, social, and sociocultural risk factors for depression have been documented (e.g., [6, 7, 8]), these dimensions are often examined separately, producing fragmented explanations of a disorder that develops through intertwined four‐dimensional processes [9, 10].

Within this multidimensional perspective, depression is consistently associated with disadvantage across biological, psychological, social, and sociocultural domains, with socioeconomic conditions especially representing a pervasive contextual influence on mental health risk. Socioeconomic disadvantage consistently predicts depression. Individuals with lower income or education are almost twice as likely to experience depressive symptoms as those with higher socioeconomic status [11, 12]. Such socioeconomic disparities also shape exposure to downstream risk factors, including limited access to healthcare, chronic stress, and constrained coping resources, which together heighten vulnerability to depression across the life course. These disparities, compounded with biological factors such as chronic illness and functional disability, further increase vulnerability to depression and reinforce symptom severity through pathways including pain, fatigue, and reduced functional capacity [13, 14]. Social isolation and loneliness are also linked to an increased risk of depression across diverse cultural and demographic contexts, reflecting the psychological and social consequences of sustained socioeconomic strain. Global analyses covering over 100 countries confirm these associations [15, 16]. Based on this evidence, we expected factors from each of the biological, psychological, social, and sociocultural dimensions to show unique conditional associations with depression within the multivariable networks.

Although more and more people recognize that depression is caused by the interaction of multiple dimensions, existing comprehensive approaches have not yet provided a sufficiently comprehensive or experience‐based explanation of how biological, psychological, social, and sociocultural processes jointly shape the risk of depression. A small number of integrative models have examined interactions among biological, psychological, and social factors, including developmental structural equation models and dynamic network frameworks [17, 18]. However, such efforts rarely extend to sociocultural influences, which are often treated as background characteristics rather than as integral components of explanatory models. As a result, broader structural conditions that shape exposure to stressors, access to resources, and cultural meaning systems remain insufficiently incorporated into empirical accounts of depression.

These fragmented lines of evidence point to the need for an integrative explanatory framework. Sue’s [19] Multipath Model of Mental Disorders conceptualizes psychopathology as the product of dynamic interactions among biological, psychological, social, and sociocultural processes. Although this framework is widely applied in education and clinical training, it has received limited empirical examination using cross‐national population data. This limitation is important because framework integration and cross‐national comparison are closely related. If depression is shaped by multiple interacting systems, then the relative importance of these systems may depend on broader institutional and cultural contexts.

Cross‐national comparison is important because the mental health consequences of income, family structure, and loneliness may depend on welfare systems, health care access, and cultural expectations of family and social support. The United Kingdom, the United States, and China provide useful contrasts in these contexts, particularly in relation to social protection, personal financial resources, and intergenerational family support [15, 20, 21]. These contrasts provide an opportunity to examine whether broadly similar depression‐related patterns emerge across cohorts and how cohort‐specific sociocultural contexts may shape these associations.

In addition, sociocultural disadvantage may not be associated with depression only through a direct relationship but also through more proximal psychological, social, and health‐related pathways. Therefore, framework integration, cross‐national comparison, and mediation analysis are complementary components of the same analytical question: whether the links between socioeconomic disadvantage and depression are shared across countries or shaped by context. To address these connected gaps, the present study applies the multipath framework as an organizing lens to identify the most influential dimensions of depression risk and examine the cross‐national consistency of these associations by modeling biological, psychological, social, and sociocultural factors in nationally representative cohorts from the United Kingdom, the United States, and China. Given the limited evidence available for precise directional predictions, we examined whether the organization and relative importance of these factors differed across national contexts. This design allows us to examine whether multipath pathways to depression are shared across contexts or context‐dependent.

### 1.1. Current Study

Building on this integrative, cross‐national application of the multipath framework, we conceptualize sociocultural factors as distal conditions that shape the social environments, psychological processes, and biological vulnerabilities through the development of depression. Empirical evidence indicates that sociocultural indicators operate as an upstream determinant of mental health, influencing depressive symptoms indirectly by structuring access to resources, exposure to chronic stress, and opportunities for social connection [22]. These sociocultural conditions may therefore shape downstream social, psychological, and biological pathways rather than exerting direct effects alone, including stress‐related physiological processes and cumulative biological burden [23, 24]. Distinguishing upstream sociocultural contexts from more proximal mechanisms is important for clarifying how depression risk is organized at the population level [25]. Accordingly, we expected lower income to be associated with greater depression and anticipated that part of this association would operate through the proposed biological, psychological, and social pathways.

This study aims to pursue three specific aims. First, we integrate biological, psychological, social, and sociocultural factors within a unified multidimensional framework to identify which dimensions are most strongly associated with depression at the population level. Second, we examine whether the relative importance of these dimensions is consistent across national contexts with distinct social and cultural systems, given the evidence that socioeconomic and ethnic inequalities in depression vary across countries [26, 27]. Third, we investigate whether the effects of sociocultural disadvantage on depression operate indirectly through social, psychological, and biological pathways, thereby clarifying the mechanisms linking structural conditions to individual mental health outcomes.

## 2. Method

### 2.1. Data Source

Secondary cross‐sectional data were obtained from participants aged 16–101 years across three large‐scale, nationally representative longitudinal surveys: the United Kingdom Household Longitudinal Study (UKHLS) [28], the Midlife in the United States Study (MIDUS) [29], and the China Health and Retirement Longitudinal Study (CHARLS) [30]. The lower age bound of 16 years was used to broadly capture the transition into working age, when labor market participation and independent income generation become possible. This threshold is consistent with international labor statistics, which commonly define the working‐age population as beginning at approximately age 15, and with the adult interview eligibility criteria of the UKHLS [28, 31].

Although participants across the three datasets ranged from 16 to 101 years, the source cohorts differed in their target populations and age distributions. UKHLS included a broader adult age range, whereas MIDUS and CHARLS were primarily designed to study middle‐aged and older adults. These differences reflect the original cohort designs rather than the different age‐based inclusion criteria applied in the present study.

The publicly available data from the UKHLS [28] represented private households across England, Scotland, Wales, and Northern Ireland. The current study used Wave 13, which was collected from January 2021 to December 2022. The interviews were conducted primarily online (computer‐assisted web interviews [CAWIs]), followed by telephone interviews (computer‐assisted telephone interviews [CATIs]). The overall household response rate was 65% among previously productive households, and the individual response rate among adults interviewed at the prior wave averaged 87%. Ethical approval for the UKHLS was granted by the University of Essex Ethics Committee on behalf of the Economic and Social Research Council (ESRC), and informed consent was obtained from all respondents before participation.

The publicly available data from the Midlife in the United States Study [29] represented U.S. residents. The current study used Wave 3, which was collected from May 2013 to November 2014. Data were collected by the University of Wisconsin Survey Center through a 45‐min CATI, a 100‐page mail self‐administered questionnaire (SAQ), and a 25‐min cognitive telephone interview. The overall response rate ranged from 77% to 83%. Ethical approval was obtained from the University of Wisconsin‐Madison Institutional Review Board, funded by the National Institute on Aging, and informed consent was obtained from all respondents before participation.

The publicly available data from the China Health and Retirement Longitudinal Study [30] represented adults living in private households across mainland China. The current study used Wave 4, which was collected in 2018. Data were collected through face‐to‐face computer‐assisted personal interviews (CAPI) with eligible respondents and their spouses. The survey included individuals living in private households such as houses and apartments and excluded institutionalized residents (e.g., hospitals, nursing homes, or correctional facilities). The initial response rate was 80.5%, and follow‐up rates exceeded 86%. Ethical approval for all CHARLS waves was obtained from the Institutional Review Board of Peking University, and informed consent was obtained from all participants before participation.

### 2.2. Measures

Detailed descriptions of variable selection, measurement instruments, and harmonization procedures are provided in Appendix A of the Supporting Information.

Variable unification across the UKHLS, MIDUS, and CHARLS datasets was conducted using consistent recoding and standardization procedures, as described in each dataset’s coding documentation. All missing or invalid values (e.g., −9 to −1) were removed prior to analysis to ensure consistency and accuracy of the sample.

Depressive symptoms (DEPR) were assessed using validated instruments in each dataset (UKHLS: GHQ‐12; MIDUS: CIDI‐SF depression module; and CHARLS: CES‐D 10). Although different instruments were used across cohorts, each has established psychometric support and has been evaluated across diverse cultural and population settings, including cross‐cultural psychometric evaluation, measurement‐invariance testing, and standardized use in international epidemiological research [32–37]. The use of cohort‐specific instruments followed the standardized assessment protocol of each nationally representative study. Measures differed across studies but were coded such that higher scores indicated greater depressive symptom severity.

In the UKHLS, depressive symptoms were measured using the 12‐item General Health Questionnaire (GHQ‐12), with items 3, 4, 7, 8, and 12 reverse‐coded and summed [32, 36, 38, 39].

In MIDUS, depressive symptoms were assessed using the depression module of the World Health Organization Composite International Diagnostic Interview Short Form (CIDI‐SF; [40]). The continuous composite score C1PDEPRE (C1DEPAF + C1PANHED; range: 0–7), which aggregates depressed affect (items C1PA60–C1PA66) and anhedonia (items C1PA72–C1PA77), was used in the present analyses. The parent CIDI has been evaluated across diverse cultural settings and standardized for use in international psychiatric epidemiological research [34, 37].

In CHARLS, depressive symptoms were assessed using the 10‐item Center for Epidemiologic Studies Depression Scale (CES‐D 10), with items 5 and 8 reverse‐coded and summed following standard scoring procedures [33, 35, 41].

### 2.3. Biological Dimension

Self‐rated health (HLTH) was assessed using a single‐item measure in each dataset (UKHLS: m_scsf1; MIDUS: C1SA1; and CHARLS: da002). Response options differed across studies but were harmonized such that higher values indicated better perceived health. In the UKHLS and CHARLS, items were reverse‐coded (UKHLS: 1 = excellent to 5 = poor; CHARLS: 1 = very good to 5 = very poor), whereas MIDUS used a 0–10 scale ranging from worse to best perceived health.

Functional disability (DISA) was assessed using items measuring limitations in activities of daily living and instrumental activities of daily living in each dataset (UKHLS: m_disdif1–m_disdif12; MIDUS: C1SA24A–C1SA24J; CHARLS: db004–db021). Response formats differed across studies but were harmonized such that higher values indicated greater levels of functional limitation. In the UKHLS, items were coded as 0 = not mentioned and 1 = mentioned (range: 0–12). In MIDUS and CHARLS, items used 1–4 scales (MIDUS: 1 = a lot to 4 = not at all; CHARLS: 1 = don’t have any difficulty to 4 = cannot do it).

Substance use (SUBS) was operationalized as the total weekly quantity of alcohol and tobacco consumption. Weekly alcohol consumption was calculated as drinking frequency multiplied by number of drinks per occasion and converted into weekly equivalents. Smoking intensity was calculated as cigarettes per day multiplied by seven, with nonsmokers coded as zero. Alcohol and tobacco variables were harmonized into weekly metrics within each cohort and summed to capture overall substance use burden. This composite approach was used to reflect cumulative exposure rather than equivalence in measurement units. Higher scores indicated greater total weekly substance use. In the UKHLS, smoking intensity was calculated as m_ncigs (cigarettes per day) × 7, with participants coded as zero if m_smoker indicated nonsmoking. Alcohol use was derived from m_auditc3 (drinking frequency) and m_auditc4 (drinks per occasion), which were recoded into weekly equivalents prior to multiplication. In MIDUS, smoking intensity was based on C1PA44 (current cigarettes per day), with nonsmokers coded as zero. Alcohol use was calculated as A1PA51 (frequency) × A1PA52 (drinks per occasion). In CHARLS, the smoking intensity was derived from da063 (cigarettes per day). Alcohol use was calculated using frequency and drinks‐per‐occasion items (da072–da077), which were recoded into weekly equivalents prior to multiplication.

### 2.4. Psychological Dimension

Positive affect (PAFF) was assessed using a single‐item measure in each dataset (UKHLS: Depress_12; MIDUS: C1SA22C; and CHARLS: dc016). Response options differed across studies but were harmonized such that higher values indicated a greater positive affect. In the UKHLS and MIDUS, items were reverse‐coded (UKHLS: 1 = more so than usual to 4 = much less than usual; MIDUS: 1 = all of the time to 5 = none of the time), whereas CHARLS used a 1–4 scale ranging from 1 = rarely or none (<1 day) to 4 = most of the time (5–7 days).

Life satisfaction (LSAT) was assessed using a single‐item measure in each dataset (UKHLS: m_sclfsato; MIDUS: C1SSATIS; and CHARLS: dc028). Response options differed across studies but were harmonized such that higher values indicated greater life satisfaction. In the UKHLS, responses ranged from 1 = completely dissatisfied to 7 = completely satisfied. In MIDUS, responses ranged from 0 = the worst possible life to 10 = the best possible life. In CHARLS, items were reverse‐coded (1 = completely satisfied to 5 = not at all satisfied).

Loneliness (LONE) was assessed using either a short multi‐item scale or a single‐item measure depending on dataset availability (UKHLS: m_sclackcom, m_scleftout, m_scisolate; MIDUS: C1SA20G; and CHARLS: dc017). Response options differed across studies but were harmonized such that higher values indicated greater perceived loneliness. In the UKHLS, loneliness was calculated as the mean of three items rated from 1 = hardly ever or never to 3 = often. In MIDUS, the item was reverse‐coded (1 = a lot to 5 = not at all). In CHARLS, responses ranged from 1 = rarely or none (<1 day) to 4 = most of the time (5–7 days).

### 2.5. Social Dimension

Marital status (MARR) was coded dichotomously as partnered versus not partnered in each dataset (UKHLS: m_marstat; MIDUS: C1PB19; and CHARLS: be001). Response categories differed across studies but were harmonized such that partnered individuals were coded as 1 and all others as 0. In the UKHLS, participants who were married or in a civil partnership were coded as partnered. In MIDUS, only those currently married were coded as partnered. In CHARLS, individuals who were married (living with or not living with spouse) were coded as partnered.

Number of children (KIDS) was calculated as the total number of living biological, adopted, and stepchildren in each dataset (UKHLS: m_nadoptch, m_nnatch; MIDUS: C1PC2; and CHARLS: xchildalive_1_–xchildalive_16_). In the UKHLS, the number of children was computed as the sum of biological and adoptive/stepchildren reported in the household. In MIDUS, the total number of living children was derived directly from C1PC2. In CHARLS, respondents provided information for each child in a roster, and the total number of living children was calculated by summing across child entries.

Family support (FSUP) was operationalized as the frequency of contact with parents in each dataset (UKHLS: m_masee, m_macon, m_pasee, m_pacon; MIDUS: C1SJ1–C1SJ2; and CHARLS: cd002_w4_1_–cd002_w4_8_). Contact frequencies were converted into weekly equivalents to ensure cross‐national comparability, and participants with no living parents were coded as zero contact frequency. In the UKHLS, the frequency of in‐person and remote contact with mother and father was converted to weekly equivalents and summed across items. In MIDUS, contact frequency with parents (in person and via phone/mail or social media) was converted into weekly equivalents and summed. In CHARLS, respondents reported the frequency of visiting parents, and weekly equivalents were calculated by summing across parental contact items.

### 2.6. Sociocultural Dimension

Personal income (INCM) was calculated as annual individual income from all available sources in each dataset (UKHLS: m_fimnnet_dv; MIDUS: C1SRINC; CHARLS: ga002, ga003_w4_1–ga003_w4_9). In the UKHLS, the monthly net personal income was multiplied by 12 to obtain the annual income. In MIDUS, annual income was derived directly from reported wages, pensions, social security, and other sources. In CHARLS, total annual income was calculated by summing the wage, pension, and other reported income sources. Income was standardized (*z*‐scored) within each cohort to reduce scale heterogeneity and improve numerical stability in mediation and network analyses.

Educational attainment (EDUY) was converted into years of schooling to ensure cross‐national comparability across datasets (UKHLS: m_qfhigh_dv; MIDUS: C1PB1; CHARLS: bd001_w2_4). Response categories differed across studies but were harmonized into estimated years of completed education following international classification conventions. In the UKHLS, the highest qualification levels were recoded into years of schooling ranging from 11 years (secondary qualifications) to 18 years (higher degree). In MIDUS, educational attainment categories were recoded into years ranging from 6 years (no formal schooling) to 20 years (doctoral or professional degree). In CHARLS, educational categories were recoded into years ranging from 0 years (no formal education) to 21 years (doctoral degree).

Racial/ethnic minority status (RMIN) was coded dichotomously according to national demographic classification systems in each dataset (UKHLS: m_racel_dv; MIDUS: C1PF7A; and CHARLS: bg001_w4). Categories differed across countries but were harmonized such that majority group members were coded as 0 and minority group members as 1 within each national context. In the UKHLS, individuals identifying as White British, Irish, or other White backgrounds were coded as majority, and all other racial/ethnic groups were coded as minority. In MIDUS, respondents identifying as White were coded as majority, and all other racial/ethnic categories were coded as minority. In CHARLS, Han nationality was coded as majority, and all other officially recognized ethnic groups were coded as minority.

### 2.7. Sample

The final analytic sample included 21,712 participants from the UKHLS, 1563 participants from MIDUS, and 5277 participants from CHARLS after excluding cases with missing data on study variables (complete case analysis). In the UKHLS, 44.4% of participants were male and 55.6% were female. In MIDUS, 35.5% were male and 64.5% were female. In CHARLS, 48.7% were male and 51.3% were female.

### 2.8. Data Analysis

SPSS 30.0 was used for descriptive statistical analysis in this study. Means, medians, standard deviations, and frequencies were calculated for all major variables across the four dimensions (biological, psychological, social, and sociocultural) as well as for depression. Age and sex were organized and summarized to describe the sample characteristics, but these demographic variables were included only as a demonstration of the data structure and were not analyzed further.

Mediation analysis used PROCESS MACRO (Version 5; [42]) to examine the mediation model and estimate indirect effects among the four dimensions. Model 4 was adopted to test whether the effects of sociocultural factors (income, education, and minority status) on depression were mediated through social, psychological, and biological dimensions. The primary objective was to compare the distinct indirect associations through loneliness, disability, and the number of children and to identify the pathway with the largest relative contribution. A parallel mediation model was selected because the cross‐sectional data did not provide a sufficient basis for establishing temporal ordering among the mediators. The resulting estimates were therefore interpreted as cross‐sectional indirect associations rather than causal sequences [43, 44].

The decision to prioritize the sociocultural dimension as the most distal predictor was based on both theoretical reasoning and empirical evidence. Extensive work in social psychiatry demonstrates that social–cultural determinants like income, education, and ethnicity shape exposure to stressors, access to resources, and cultural meaning systems that contextualize individual experiences of depression [11, 12]. However, compared to biological, psychological, and interpersonal mechanisms, relatively few studies examine these broader sociocultural contexts [45, 46]. Recent umbrella and systematic reviews further note that although social determinants of health are increasingly recognized, sociocultural dimensions, such as institutional inequality, minority stress, and educational disadvantage, are among the least investigated pathways in comprehensive depression research [47, 48]. This paucity of research supports viewing the sociocultural dimension as the distant context from which social relationships, psychological processes, and biological responses develop, finally influencing vulnerability to depression. Indirect effects were tested using the bootstrap method (5000 resamples), and 95% bias‐corrected confidence intervals were computed. When zero was not included in the confidence interval, the indirect effect was considered statistically significant.

The network analysis was performed in RStudio Version 2025.05.1 (Posit Software, PBC) with R Version 4.5.1. First, the “*qgraph*” package was used to establish the network, which was estimated using a Gaussian graphical model based on the Extended Bayesian Information Criterion Graphical LASSO (EBICglasso) method. This method applies LASSO regularization to obtain a sparse structure and selects the optimal model according to the EBIC criterion [49, 50]. EBICglasso was selected because it can estimate conditional relationships among variables and also reduce small or unstable edges in the network. In this model, edges represent partial correlations between two variables after controlling for all other variables in the network. The tuning parameter γ determines how strongly model complexity is penalized, with higher γ values producing more conservative and sparser networks [49]. In the main analysis, γ was estimated using the default EBICglasso tuning parameter γ = 0.50, which is commonly used in psychological network analysis [51]. This network consisted of 13 variables from the four dimensions: three biological, three psychological, three social, three sociocultural indicators, and depression.

## 3. Results

Table 1 summarizes the descriptive statistics for all continuous study variables from the UKHLS, MIDUS, and CHARLS datasets. Means and standard deviations were calculated for each continuous variable within the four dimensions (biological, psychological, social, and sociocultural) and for depression. Because the instruments and response ranges differed across datasets, these descriptive statistics are presented only to illustrate the distribution of variables within each dataset rather than for direct comparison across samples or measures. The primary purpose of this analysis was to describe data characteristics before conducting mediation and network analyses that focus on the relationships among variables rather than on absolute score differences.

**Table 1:** Descriptive statistics across cohorts.

Variables	UKHLS (*N* = 21,712) M	SD	MIDUS (*N* = 1563) M	SD	CHARLS (*N* = 5277) M	SD
Age	50.684	18.312	63.120	10.788	62.642	9.819
Depression	23.678	5.695	0.629	1.728	20.196	6.889
Self‐rate health	3.265	1.0188	7.275	1.563	2.809	0.954
Substance use	12.528	36.541	22.690	64.185	45.720	89.102
Functional disability	0.582	1.328	18.281	8.797	21.129	6.040
Loneliness	4.437	1.673	1.552	0.863	1.774	1.115
Positive affect	2.868	0.554	2.900	0.955	2.830	1.202
Life satisfaction	3.165	0.833	7.724	1.277	3.165	0.833
Family support	5.622	9.135	8.871	7.663	1.282	2.769
Number of children	0.691	1.034	2.422	1.671	2.862	1.584
Personal income	22,790.545	28,338.004	56,196.737	58,158.240	9846.079	25577.052
Education	11.424	6.0247	14.580	2.437	5.286	4.049

Abbreviations: M, mean; SD, standard deviation.

### 3.1. Stability

**Figure 1:**
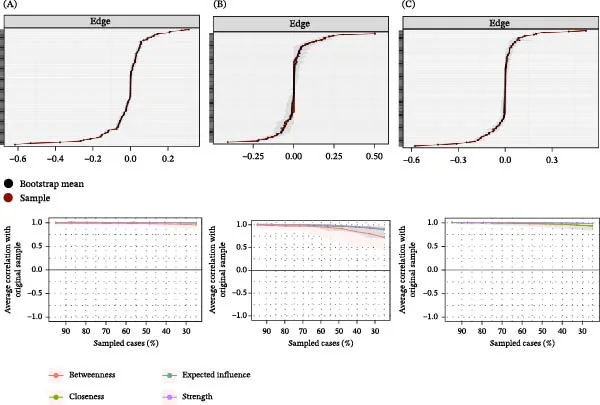
Edge‐weight accuracy and centrality stability across the (A) UKHLS, (B) MIDUS, and (C) CHARLS networks.

The case‐dropping *bootstrap* results showed high stability across all three national cohorts (more details are given in Appendix A of the Supporting Information). As shown in Figure 1, the average correlations between the bootstrapped and original centrality indices remained above 0.9 for all four measures through the resampling procedure.

In the UKHLS and CHARLS networks, all indices remained above 0.95 even when 70% of the cases were dropped, yielding centrality stability coefficients of approximately CS [cor = 0.7] = 0.75, which exceeds the recommended threshold of 0.5 [52]. The MIDUS network also showed strong stability, with correlations above 0.9 for strength, closeness, and expected influence, though betweenness exhibited a slightly larger decline at higher levels of case removal. Overall, these results confirm that the centrality estimates across all three networks were highly robust and reliable. Shaded areas in Figure 1 represent 95% confidence intervals. Edge‐weight difference tests are shown in Figure S1.

### 3.2. Mediation Analysis

**Figure 2:**
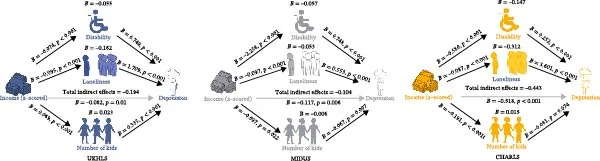
Mediation analysis across UKHLS, MIDUS, and CHARLS. UKHLS, Understanding Society: The UK Household Longitudinal Study; MIDUS, Midlife in the United States; CHARLS, China Health and Retirement Longitudinal Study.

Figure 2 presents the parallel mediation model, in which all paths were statistically significant in most cohorts. Specifically, income exerted a significant indirect effect on depression through disability, loneliness, and the number of children. The pathway from the number of children to depression was not statistically significant in the CHARLS cohort. Among these mediators, loneliness demonstrated the strongest and most consistent mediating effects across the UKHLS, MIDUS, and CHARLS cohorts. The total indirect effects were −0.194 for UKHLS, −0.104 for MIDUS, and −0.443 for CHARLS, suggesting that the indirect association between lower income and higher depression was largely attributable to loneliness and disability across all three cohorts.

### 3.3. Network Analysis

**Figure 3:**
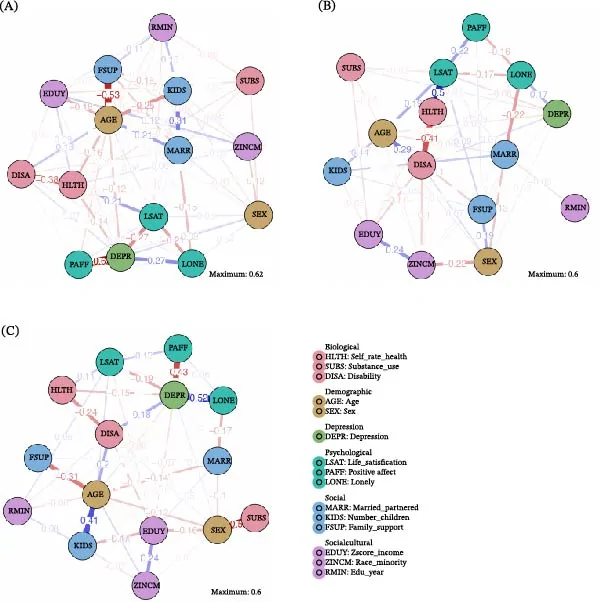
Network structures across (A) UKHLS, (B) MIDUS, and (C) CHARLS. Note: Networks were estimated using the EBICglasso model based on Gaussian graphical models (GGMs) with standardized continuous and binary variables. Each node represents one observed variable, and edges reflect partial correlations after controlling for all other variables. Blue edges indicate positive associations, red edges indicate negative associations, and thicker lines correspond to stronger correlations.

Figure 3A presents the EBICglasso partial correlation network in the UKHLS. Depression (DEPR) showed a strong negative association between positive affect (PAFF, *r* = −0.62) and life satisfaction (LSAT, *r* = −0.27), indicating that lower positive emotions and reduced life satisfaction were closely related to higher depressive symptoms. In contrast, DEPR was positively associated with loneliness (LONE, *r* = 0.27), suggesting that social isolation was important to depression. Moderate negative connections were also observed with self‐rated health (HLTH, *r* = −0.14) and age (AGE, *r* = −0.12), implying that poorer perceived health and older age were linked to greater depressive symptoms.

Figure 3B presents the EBICglasso partial correlation network in the MIDUS cohort. Depression (DEPR) showed a positive association with loneliness (LONE, *r* = 0.18), indicating that greater social isolation was related to higher depressive symptoms. No other connections exceeded the |*r*| ≥ 0.10 threshold, suggesting that loneliness was the primary correlate of depression in the MIDUS network.

Figure 3C presents the EBICglasso partial correlation network in the CHARLS cohort. Depression (DEPR) showed strong negative associations with positive affect (PAFF, *r* = −0.43) and life satisfaction (LSAT, *r* = −0.19), indicating that lower positive emotions and reduced life satisfaction were related to higher depressive symptoms. In contrast, DEPR was positively associated with loneliness (LONE, *r* = 0.52) and disability (DISA, *r* = 0.18), suggesting that social isolation and physical impairment were important correlates of depression. A moderate negative association was also observed with self‐rated health (HLTH, *r* = −0.15), implying that poorer perceived health was linked to higher depressive symptoms.

Centrality analysis (Figure 4A) showed that age (AGE) and depression (DEPR) ranked highest on the centrality indices. According to the metrics, AGE and DEPR had the greatest strength, closeness was next highest for AGE and self‐rated health (HLTH), expected influence was next highest for married/partnered (MARR), education years (EDUY), number of children (KIDS), race minority (RMIN), and sex (SEX), and betweenness was next highest for AGE and DEPR. These patterns indicate that AGE and DEPR are the dominant hubs in the UKHLS network, with HLTH, MARR, EDUY, KIDS, RMIN, and SEX contributing secondary central roles [52, 53].

Figure 4Node centrality indices across (A) UKHLS, (B) MIDUS, and (C) CHARLS.

**Figure:**
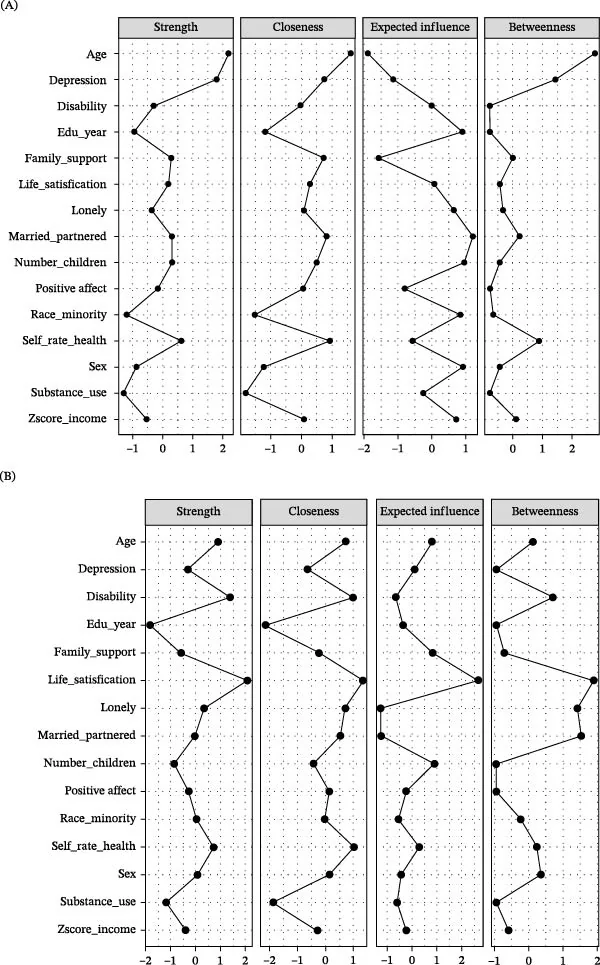

**Figure:**
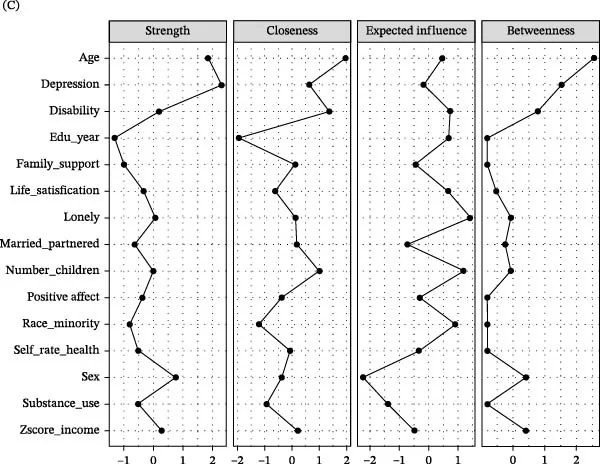

Centrality analysis (Figure 4B) showed that life satisfaction (LSAT) ranked highest on all four indices. According to the metrics, Disability (DISA) was second in strength, and closeness was next highest for disability (DISA) and self‐rated health (HLTH). Expected influence was next highest for age (AGE), family support (FSUP), and number of children (KIDS), and betweenness was next highest for number of children (KIDS), followed by loneliness (LONE). These patterns indicate that LSAT is the dominant factor and bridge in the MIDUS network, with DISA, HLTH, AGE, FSUP, KIDS, and LONE contributing secondary central roles [52, 53].

Centrality analysis (Figure 4C) showed that depression (DEPR) and age (AGE) had the highest strength, indicating that they were the most strongly connected nodes within the CHARLS network. Closeness was next highest for age (AGE) and disability (DISA), suggesting that these nodes were most proximal to others and served as key connectors in the network. Expected influence was comparatively high for loneliness (LONE), number of children (KIDS), and race minority (RMIN), reflecting their broad overall impact across domains. Betweenness was the highest for age (AGE) and depression (DEPR), implying that they frequently bridged indirect connections between other nodes. Taken together, these findings indicate that Age and Depression were the dominant nodes of the CHARLS network, with disability, loneliness, number of children, and race minority contributing secondary central roles [52, 53].

Detailed centrality metrics are available in the Supporting Information (Figures S2–S4).

## 4. Discussion

This study tested Sue’s [19] multipath model of mental disorders by examining how biological, psychological, social, and sociocultural factors together influence depression in three national cohorts. The results supported the model and showed that all four dimensions were related to depression. However, the psychological dimension showed the strongest and most consistent effect, especially through loneliness, positive affect, and life satisfaction. These results support the idea that mental disorders come from the interaction of different systems, but the strength of each system’s influence is not equal [19, 54].

Beyond supporting the multipath model, the present study also demonstrates the value of integrating complementary analytical approaches to understanding depression risk. Previous large‐scale research has examined the associations between socioeconomic inequality and depressive disorders using regression‐based approaches, including mixed‐effects and linear regression models across 122 countries [55]. The present study extends this association‐focused approach by combining mediation and network analyses. Mediation analysis was used to examine whether sociocultural disadvantage was associated with depression indirectly through biological, psychological, and social dimensions. This pathway‐level finding is important because it points out that, other than immediately increasing income itself, which rarely happens, loneliness may therefore serve as a practical intervention target for reducing depression risk among individuals experiencing socioeconomic disadvantage. Network analysis added a second level of contribution by showing that these factors were better understood not simply as isolated correlates of depression but as components of an interconnected system of depression‐related risks. In particular, centrality patterns helped identify which variables occupied more influential positions in the system. This information complements conventional regression‐based models, which estimate associations between specified predictors and outcomes but do not directly characterize the broader pattern of interrelations among risk factors [56]. Together, these findings show that a combination of mediation and network analyses can link pathway‐level explanations with system‐level organization, conceptually replicating and extending recent large‐scale population health research, showing that these two approaches can provide complementary insights into complex health outcomes [57].

Both mediation and network analyses showed that psychological factors were the main contributors to depression. In the UKHLS and CHARLS networks, positive affect and life satisfaction had the strongest negative links with depression, while loneliness was positively related to depression in all three cohorts. This finding agrees with many studies showing that lower positive emotions and well‐being predict higher depression [58, 59]. The mediation analysis also found that the feeling of social isolation may be a universal mechanism through which low income increases depression. This is in line with previous studies that identify loneliness as a key risk factor for depression and anxiety [60–62].

Despite the overall prominence of psychological factors across cohorts, the relative balance between the direct and indirect effects of income differed. This consistency between the Western samples (UKHLS and MIDUS) and the Eastern sample (CHARLS) suggests that emotional and social factors connect the broader effects of social and biological risks to depression [63, 64]. Interestingly, the relative balance between the direct and indirect effects of income differed across cohorts. In the UKHLS cohort, the total indirect effect of income on depression was larger than the direct effect, suggesting that income was more strongly related to depression through mediating pathways, especially loneliness, disability, and family structure. One possible explanation is that stronger public welfare and health protections may reduce some of the direct material consequences of low income, making psychosocial pathways more visible in the income‐depression association. This interpretation is consistent with prior research showing that social protection and welfare state spending can shape socioeconomic inequalities in depressive symptoms [20, 21]. In contrast, in the MIDUS and CHARLS cohorts, the direct effect of income remained stronger. For the MIDUS cohort, this may reflect the greater role of personal financial resources in the United States, where economic disadvantage may translate more directly into financial strain, material insecurity, and barriers to health care. This interpretation is consistent with evidence showing a stable association between financial strain and depression in the United States [65]. In the CHARLS cohort, the stronger direct effect may reflect the cultural and family context of later life in China. For older Chinese adults, income may carry meanings beyond individual economic resources, including family responsibility, perceived social status, and the ability to meet intergenerational expectations. Prior research has shown that intergenerational support is closely related to depression among Chinese older adults, suggesting that family relationships and support systems may shape how economic resources affect mental health in later life [66]. These findings show that the effects of sociocultural context are unique and context‐dependent and should be considered when explaining how income relates to depression.

Another important finding concerns the role of family structure. In the UKHLS and MIDUS cohorts, having more children was linked to lower depression, whereas in the CHARLS cohort, it was linked to higher depression. This difference may reflect how family responsibilities, intergenerational expectations, and social support systems differ across national and age‐related contexts. In Western societies, having children may provide emotional support, social connection, and a sense of meaning, which may help protect against depression [67]. In China, where family caregiving and filial piety remain strong and where later‐life support has historically depended more heavily on family networks, older adults with more children may face greater financial expectations, caregiving stress, or family conflict, which could increase depression [68]. In this context, the number of children may also reflect intergenerational transfers, multigenerational living arrangements, and expectations of family‐based care, all of which are closely tied to older adults’ psychological well‐being [69, 70].

This interpretation should also be considered in relation to the differences in elderly care systems and social security arrangements. In contexts where formal old‐age support and public care systems are more developed, children may function more as a source of emotional and social support. In contexts where later‐life support depends more heavily on family networks, children may represent not only social connection but also intergenerational obligations and care‐related stress. It is also important to note that all three cohorts included middle‐aged and older adults and that the CHARLS cohort was especially concentrated among older respondents. Therefore, these results may partly reflect age‐related differences in family roles and may not represent how younger people today view family life or the effects of having children. Number of children may also be confounded with age, marital status, health, and socioeconomic position. Therefore, this result should not be interpreted as evidence that children have the same meaning across countries. Rather, it suggests that the social dimension of mental health is shaped by culture, generation, welfare context, and intergenerational expectations.

From a theoretical perspective, these findings expand Sue’s [19] multipath model by showing that emotional well‐being and social connectedness are key psychological mechanisms through which socioeconomic and biological risks affect depression. They also support Engel’s [54] biopsychosocial model, showing that the psychological system plays an active role in linking external social and biological factors with emotional outcomes. Previous research has shown that processes such as emotion regulation [71], social support [72], and psychological well‐being [73] are central to resilience and mental health, which is consistent with our results.

From a practical point of view, the findings highlight the importance of promoting positive emotions, social connection, and reduced loneliness in preventing and reducing depression. These findings can translate into several concrete recommendations for mental health and primary care settings. Clinicians could routinely assess loneliness, perceived social isolation, positive affect, life satisfaction, mobility limitations, and difficulties in daily functioning as part of the depression risk assessment. For loneliness and social isolation, referral pathways could include community groups, peer‐support programs, volunteer activities, recreational programs, or technology‐assisted social contact. These approaches are supported by loneliness intervention research and social prescribing studies, which suggest that structured psychosocial and community‐based interventions may improve social connection and mental well‐being [74–76]. To promote positive affect and life satisfaction, brief psychological strategies such as gratitude practices, mindfulness‐based exercises, and behavioral activation may also be useful as positive psychology interventions have shown benefits across different cultural contexts [77, 78]. For patients with functional disability, chronic health problems, or difficulties in daily functioning, depression treatment could be combined with rehabilitative support. Rehabilitative support includes physical or occupational therapy referral, mobility and fall‐risk assessment, activity pacing, self‐management planning, and care coordination in primary care. This is consistent with adult clinical guidance for depression in the context of chronic physical health problems and rehabilitation frameworks that emphasize improving functioning and reducing disability [79, 80].

At the policy level, these findings suggest that reducing income‐related depression risk may require targeted improvements to existing national policies. In the United Kingdom, incorporating stronger mental health safeguards and reducing administrative burdens within universal credit may help prevent the income‐support process itself from contributing to psychological distress [81]. In the United States, expanding predictable and refundable income support, such as monthly Child Tax Credit payments for low‐income families, may help reduce the financial strain associated with depressive symptoms [82]. In China, increasing public financing and the benefit level of the rural basic pension, particularly for low‐income older adults, may strengthen financial security and reduce late‐life depression risk [83]. These recommendations illustrate how existing policies could be adjusted to address the income‐related mechanisms identified in each cohort.

Policy and clinical interventions should also be adapted to the sociocultural context in which income‐related depression risk occurs. In collectivistic cultures, family‐ or community‐based programs may be more effective, while in individualistic societies, approaches that increase autonomy and self‐efficacy may work better [84]. Overall, these findings suggest that clinical responses to depression should address psychological, social, and functional pathways within each sociocultural context.

### 4.1. Limitations and Future Direction

This study has minimized potential limitations as much as possible within the controllable scope. However, there are still several issues that need interpretation.

First, the three national cohorts differed in sampling strategies, survey instruments, and data‐coding procedures; this may cause similar variables to be measured differently across databases, reducing the accuracy of cross‐cohort comparisons. Although measurement equivalence was tested and variable harmonization was performed across cohorts, minor differences in data collection and item wording may still exist, which could introduce small inconsistencies across cohorts [85, 86]. Future research should incorporate formal tests of longitudinal and cross‐cultural measurement invariance or apply integrative data analysis frameworks that jointly model item‐level differences across cohorts.

Second, the present study relied on cross‐sectional network models in which edge weights reflect conditional associations after controlling for all other nodes; therefore, the causal direction or temporal ordering cannot be inferred. Although our analytical framework conceptualized income as an upstream socioeconomic factor associated with depression through biological, psychological, and social pathways, the reverse direction remains plausible. In particular, the social selection hypothesis suggests that psychiatric symptoms may contribute to lower income or downward socioeconomic mobility through impaired functioning, reduced employment stability, and diminished social participation [87, 88]. Consequently, the observed mediation pathways should be interpreted as cross‐sectional indirect associations rather than evidence that income causally affects depression through the proposed mediators, as cross‐sectional mediation estimates may not accurately represent longitudinal processes [44]. Future studies should collect repeated measurements of income, the proposed mediators, and depression and test the competing directions simultaneously. Random‐intercept cross‐lagged panel models could be used to examine whether within‐person changes in income predict subsequent changes in depression and whether changes in depression predict subsequent changes in income, while separating these processes from stable between‐person differences [89]. Longitudinal mediation models with temporally separated measurements could further test whether changes in income precede changes in loneliness, disability, or family related factors and whether these changes subsequently predict depression. Time‐series network models may also help identify the temporal ordering and reciprocal relationships among these factors [52].

Third, the EBICglasso model was applied to regularize the network structure. Although this method improves interpretability by yielding a sparse model, it may shrink small but potentially meaningful edges to zero, leading to the omission of weak associations [50, 52]. Examining the stability of findings across alternative regularization strategies may help clarify the robustness of these associations.

Fourth, the present analyses modeledthe income using linear associations. However, prior research suggests that the relationship between income and mental health may be nonlinear, with income having different implications at lower versus higher levels of the income distribution [90]. Future studies should examine nonlinear specifications, threshold effects, or spline‐based models to test whether the income‐depression association varies across income levels.

Finally, the three cohorts differed in their age structures, which may affect the interpretation of cross‐cohort findings. Although the lower age bound of 16 years was used to broadly capture the transition into potential labor market participation and independent income generation, the UKHLS covered a wider adult age range, whereas MIDUS and CHARLS were more concentrated among middle‐aged and older adults. Because age is closely associated with income, disability, family structure, and depression, these differences may have contributed to some of the observed cohort‐specific patterns, particularly those involving the number of children and functional disability. Although age was included in the analytical models, statistical adjustment may not fully account for differences in age composition. The findings should therefore be interpreted as comparisons across cohort‐specific adult samples rather than fully age‐equivalent national populations. Future studies should use overlapping age ranges or age‐stratified analyses to examine whether the identified pathways differ across stages of adulthood.

Overall, this study controlled for methodological differences and statistical biases as rigorously as possible across databases and cultural settings. However, the identified limitations should be considered when interpreting the main findings. Differences in depression measurement may limit direct comparability across cohorts. Differences in age structure may also contribute to cross‐national differences in family structure, disability, and depression. In addition, the cross‐sectional design prevents the indirect effects from being interpreted as causal mediation pathways. Therefore, the findings should be interpreted as cohort‐specific association patterns rather than definitive evidence of cross‐nationally equivalent or causal mechanisms. Future research should include harmonized depression measures, more comparable age structures, measurement invariance testing, multiple imputation or robust estimation techniques, complex survey weighting, and longitudinal or time‐series network analyses to enhance the robustness and clarity of the findings.

## 5. Conclusion

In summary, this study provides cross‐cohort evidence consistent with Sue’s [19] multipath model of mental disorders. Across the three national cohorts, depression was associated with biological, psychological, social, and sociocultural factors. The psychological domain, particularly loneliness, positive affect, and life satisfaction, showed the strongest and most consistent links to depression. Biological, social, and sociocultural factors also played important roles, although their associations with depression varied across cohorts and were often expressed through indirect pathways. These findings highlight the central importance of psychological well‐being and social connection in the income‐depression relationship while also showing that this relationship depends on broader sociocultural and institutional contexts. Overall, the results suggest that efforts to reduce depression should combine psychological and social interventions, functional support, and culturally sensitive policy approaches.

## Author Contributions

**Rachel Li**: conceptualization, methodology, formal analysis, investigation, resources. **Rachel Li and Xiantong Yang:** writing – original draft preparation, writing – review and editing. **Xiantong Yang**: supervision.

## Funding

The authors have nothing to report.

## Disclosure

All authors have approved the manuscript for publication. Rachel Li would like to declare on behalf of my coauthor that the work described was original research that has not been published previously and not under consideration for publication elsewhere, in whole or in part. All the authors listed have approved the manuscript.

## Ethics Statement

The present study involved secondary analysis of deidentified data and did not involve direct contact with human participants.

## Consent

All participants were informed that the survey was voluntary and anonymous before filling in the questionnaire. They were told that they could quit at any time.

## Conflicts of Interest

The authors declare no conflicts of interest.

## Supporting Information

Additional supporting information can be found online in the Supporting Information section.

## Supporting information


**Supporting Information** Detailed descriptions of variable selection, measurement instruments, and harmonization procedures are provided in Appendix A of the Supporting Information. The Supporting Information also include additional robustness analyses, including edge‐weight difference tests and node centrality estimates across the UKHLS, MIDUS, and CHARLS networks. Supporting Figures S1–S4 present these additional results.

## Data Availability

The analysis code and cleaned data supporting the findings of this study are available on the Open Science Framework (OSF) repository at https://osf.io/phj65/overview?view_only=62b56efeb8a9457f9ff00be3ab3fb901. The raw datasets used in this study were obtained from publicly available data repositories, including the UK Household Longitudinal Study (UKHLS), the Midlife in the United States Study (MIDUS), and the China Health and Retirement Longitudinal Study (CHARLS). Due to data use agreements, the raw data cannot be redistributed by the authors, but can be accessed directly from the respective data providers upon reasonable request and registration.
