# Supplementary material for: The Associations Between Income and Depression: A Cross‐National Analysis
Source: Depress Anxiety. 2026 Aug 21;2026:5511191. doi: 10.1155/da/5511191 (PMC13495565; doi:10.1155/da/5511191)
Supplement: Supplementary file 1 [file DA-2026-5511191-s001.zip › Supplementary Materials/5511191.f1.docx]

Appendix A. Detailed Description of Measures and Supplementary Analyses

All study variables were selected and harmonized based on the Multipath Model of Mental Disorders (Sue et al., 2022), and three standardized indicators represented each dimension. Each variable was chosen based on empirical evidence and supported by validated measurement methods commonly used in epidemiological and cross-national research.

**Depression (DEPR)**

Depressive symptoms (DEPR) were assessed using validated instruments in each dataset (UKHLS: GHQ-12; MIDUS: CIDI-SF depression module; CHARLS: CES-D 10). Although different instruments were used across cohorts, each has established psychometric support and has been evaluated across diverse cultural and population settings, including cross-cultural psychometric evaluation, measurement-invariance testing, and standardized use in international epidemiological research (Gelaye et al., 2015; González et al., 2017; Kessler & Üstün, 2004; Mohebbi et al., 2018; Romppel et al., 2017; Wittchen et al., 1991). The use of cohort-specific instruments followed the standardized assessment protocol of each nationally representative study. Measures differed across studies but were coded such that higher scores indicated greater depressive symptom severity.

In the UKHLS, depressive symptoms were measured using the 12-item General Health Questionnaire (GHQ-12), with items 3, 4, 7, 8, and 12 reverse-coded and summed (Gelaye et al., 2015; Goldberg & Williams, 1988; Lundin et al., 2016; Romppel et al., 2017).

In MIDUS, depressive symptoms were assessed using the depression module of the World Health Organization Composite International Diagnostic Interview Short-Form (CIDI-SF; Kessler et al., 1998). The continuous composite score C1PDEPRE (C1DEPAF + C1PANHED; range: 0-7), which aggregates depressed affect (items C1PA60-C1PA66) and anhedonia (items C1PA72-C1PA77), was used in the present analyses. The parent CIDI has been evaluated across diverse cultural settings and standardized for use in international psychiatric epidemiological research (Kessler & Üstün, 2004; Wittchen et al., 1991).

In CHARLS, depressive symptoms were assessed using the 10-item Center for Epidemiologic Studies Depression Scale (CES-D 10), with items 5 and 8 reverse-coded and summed following standard scoring procedures (Andresen et al., 1994; González et al., 2017; Mohebbi et al., 2018).

**Biological Dimension**

**Self-Rated Health (HLTH)**

Self-rated health captures an individual’s overall perception of physical well-being, integrating information about chronic illnesses, physical functioning, and symptom burden. Poor self-rated health is associated with an increased risk of depression across populations and age groups (Idler & Benyamini, 1997).

Self-rated health was derived from a single item in each dataset (UKHLS: m_scsf1; MIDUS: C1SA1; CHARLS: da002). Single-item self-rated health measures are reliable predictors of morbidity and depressive symptoms in large epidemiologic studies (Jylhä, 2009).

**Functional Disability (DISA)**

Functional disability was included as an indicator of biological vulnerability, reflecting underlying physiological decline and limitations in activities of daily living (ADL) and instrumental activities of daily living (IADL). Functional limitations are well-established contributors to later-life depression and central to the disablement process model (Katz et al., 1963; Lawton & Brody, 1969; Zhu et al., 2024).

Disability scores were computed by summing ADL- and IADL-like items across datasets (UKHLS: m_disdif1- m_disdif12; MIDUS: C1SA24A-J; CHARLS: db004-db021), with higher scores indicating greater disability. This summed approach is widely used in gerontology and psychiatric epidemiology (Prince et al., 1997).

**Substance Use (SUBS)**

Substance use included alcohol and tobacco consumption, which affect neurobiological systems involved in depression, such as inflammation and reward circuitry (Boden & Fergusson, 2011; Fluharty et al., 2017).

Weekly alcohol consumption was calculated as frequency multiplied by drinks per occasion, converted to weekly equivalents. Smoking intensity was calculated as cigarettes per day multiplied by seven, with non-smokers coded as zero. These calculations follow the Quantity-Frequency (Q×F) method for alcohol exposure (Greenfield, 2000) and standard metrics for smoking behavior (Centers for Disease Control and Prevention, 2010). Alcohol and nicotine were selected as they are legal substances in all three countries and provide comparable indicators of substance use behavior (Luger et al., 2014).

**Psychological Dimension**

**Positive Affect (PAFF)**

Positive affect was included as a psychological indicator because reduced capacity for pleasure (anhedonia) is a core feature of depression. Empirical studies report a moderate inverse association between positive affect and depressive symptoms in clinical populations (Schürmann-Vengels et al., 2025), and longitudinal evidence indicates that dampening of positive affect predicts subsequent depressive states (Bean et al., 2022).

Positive affect was operationalized using a single-item indicator of happiness (UKHLS: GHQ-12 item 12; MIDUS: C1SA22C; CHARLS: CES-D item 8), with higher scores indicating greater positive affect.

**Life Satisfaction (LSAT)**

Life satisfaction reflects the cognitive component of subjective well-being. Lower life satisfaction is associated with higher depressive symptoms and poorer recovery trajectories (Cheung & Lucas, 2014; Koivumaa-Honkanen et al., 2004).

Life satisfaction was measured using a single-item Likert scale in each dataset (UKHLS: m_sclfsato; MIDUS: C1SSATIS; CHARLS: dc028). Single-item life satisfaction measures are highly correlated with multi-item scales such as the Satisfaction With Life Scale and demonstrate good reliability (Pavot & Diener, 1993).

**Loneliness (LONE)**

Loneliness was conceptualized as perceived social isolation, a major psychological risk factor for depression. Theoretical models suggest that loneliness increases depressive vulnerability through maladaptive emotion regulation and social cognition biases (Cacioppo et al., 2010).

Loneliness was measured using either the mean of three short items (UKHLS: m_sclackcom, m_scleftout, m_scisolate) or a single-item indicator (MIDUS: C1SA20G; CHARLS: CES-D item 9). Short-form UCLA Loneliness Scale items are widely used in population surveys and show robust associations with depressive symptoms (Hughes et al., 2004; Mund & Neyer, 2019).

**Social Dimension**

**Marital Status (MARR)**

Marital status was included as a social indicator because marriage and cohabitation provide emotional security and instrumental support that buffer against stress. Prior research consistently shows lower depressive symptoms among partnered individuals compared with those who are single, divorced, or widowed (Simon & Barrett, 2010; Strohschein et al., 2005; Williams & Umberson, 2004).

Marital status was coded dichotomously as married or cohabiting versus not married (UKHLS: m_marstat; MIDUS: C1PB19; CHARLS: be001).

**Number of Children (KIDS)**

The number of children reflects family network size and potential intergenerational support. Parenthood can expand social connections and emotional purpose but may also introduce role strain depending on life stage and cultural context (Nomaguchi & Milkie, 2020; Umberson et al., 2010).

The total number of living biological, adopted, and stepchildren was counted in each dataset (UKHLS: m_nnatch + m_nadoptch; MIDUS: C1PC2; CHARLS: cb050_w3).

**Family Support (FSUP)**

Family support was operationalized using frequency of contact with parents, reflecting connectedness and perceived availability of emotional and instrumental support. In many cultures, parent-child ties remain an important source of support throughout adulthood (Thoits, 2011).

Contact frequencies were converted into weekly equivalents to ensure comparability across datasets (UKHLS: m_masee, m_macon, m_pasee, m_pacon; MIDUS: C1SJ1–C1SJ2; CHARLS: cd002). Participants with no living parents were coded as zero. This approach follows procedures commonly used in social network research, including the Lubben Social Network Scale (Lubben et al., 2006).

**Sociocultural Dimension**

**Personal Income (INCM)**

Income was treated as a structural indicator of socioeconomic position, reflecting access to material resources and exposure to financial stress. Lower income is consistently associated with higher rates of depressive symptoms and poorer psychological well-being (Lorant et al., 2003; Ridley et al., 2020).

Income was standardized across datasets as annual personal income derived from wages, pensions, and other monetary sources (UKHLS: m_fimnnet_dv × 12; MIDUS: C1SRINC; CHARLS: sum of pension and wage sources).

**Education (EDUY)**

Education reflects long-term access to knowledge, cognitive resources, and opportunities, as well as position within social hierarchies. Lower educational attainment is associated with higher depressive symptoms and reduced help-seeking (Bjelland et al., 2008).

Educational attainment was converted into years of schooling following international classification guidelines to ensure cross-national comparability (UKHLS: m_qfhigh_dv; MIDUS: C1PB1; CHARLS: bd001_w2_4).

**Racial/Ethnic Minority Status (RMIN)**

Minority status reflects exposure to structural inequities and cultural marginalization. Individuals identifying as racial or ethnic minorities experience greater exposure to chronic stressors and barriers to culturally responsive mental-health care, elevating depression risk (Ahmad et al., 2021; Williams et al., 2019).

Minority status was coded according to national demographic contexts, with non-White participants coded as minorities in the United Kingdom and the United States, and non-Han participants coded as minorities in China (UKHLS: m_racel_dv; MIDUS: C1PF7A; CHARLS: bg001_w4). Although ethnicity itself is not a direct cause of depression, its intersection with socioeconomic disadvantage and institutional inequities makes it a key sociocultural risk factor.

Figure S1. Edge-weight difference test results for the UKHLS, MIDUS, and CHARLS networks

Figure S2. Node centrality metrics (strength, closeness, expected influence, and betweenness) in the UKHLS network

Figure S3. Node centrality metrics (strength, closeness, expected influence, and betweenness) in the MIDUS network

Figure S4. Node centrality metrics (strength, closeness, expected influence, and betweenness) in the CHARLS network

**References**

Ahmad, G., McManus, S., Cooper, C., Hatch, S., & Das-Munshi, J. (2021). Prevalence of common mental disorders and treatment receipt for people from ethnic minority backgrounds in England: Repeated cross-sectional surveys of the general population in 2007 and 2014. *The British Journal of Psychiatry*. <https://doi.org/10.1192/bjp.2021.179>

American Psychiatric Association. (2013). Diagnostic and statistical manual of mental disorders (5th ed.). American Psychiatric Publishing.

Andresen, E. M., Malmgren, J. A., Carter, W. B., & Patrick, D. L. (1994). Screening for depression in well older adults: Evaluation of a short form of the CES-D. *American Journal of Preventive Medicine, 10(2)*, 77–84.

Bean, C. A. L., Summers, C., & Ciesla, J. A. (2022). Dampening of positive affect and depression: A meta-analysis of cross-sectional and longitudinal relationships. *Behaviour Research and Therapy, 156*, 104153. <https://doi.org/10.1016/j.brat.2022.104153>

Bjelland, I., Krokstad, S., Mykletun, A., et al. (2008). Does a higher educational level protect against anxiety and depression? The HUNT study. *Social Science & Medicine, 66*(6), 1334–1345. <https://doi.org/10.1016/j.socscimed.2007.12.019>

Boden, J. M., & Fergusson, D. M. (2011). Alcohol and depression. *Addiction, 106*(5), 906–914. <https://doi.org/10.1111/j.1360-0443.2010.03351.x>

Cacioppo, J. T., Hawkley, L. C., & Thisted, R. A. (2010). Perceived social isolation makes me sad: Five-year cross-lagged analyses of loneliness and depressive symptomatology in older adults. *Psychology and Aging, 25*(2), 453–463. <https://doi.org/10.1037/a0017216>

Centers for Disease Control and Prevention. (2010). How tobacco smoke causes disease: The biology and behavioral basis for smoking-attributable disease: A Report of the Surgeon General. U.S. Department of Health and Human Services, Centers for Disease Control and Prevention. Retrieved from [https://www.ncbi.nlm.nih.gov/books/NBK53017/](https://www.ncbi.nlm.nih.gov/books/NBK53017/?utm_source=chatgpt.com)

Cheung, F., & Lucas, R. E. (2014). Assessing the validity of single-item life satisfaction measures: Results from three large samples. *Quality of Life Research, 23*(10), 2809–2818. <https://doi.org/10.1007/s11136-014-0726-4>

Fluharty, M., Taylor, A. E., Grabski, M., & Munafò, M. R. (2017). The Association of Cigarette Smoking with depression and anxiety: A systematic review. *Nicotine & Tobacco Research*, *19*(1), 3–13. <https://doi.org/10.1093/ntr/ntw140>

Gelaye, B., Tadesse, M. G., Lohsoonthorn, V., Lertmeharit, S., Pensuksan, W. C., Sanchez, S. E., Lemma, S., Berhane, Y., Vélez, J. C., Barbosa, C., Andrade, A., & Williams, M. A. (2015). Psychometric properties and factor structure of the General Health Questionnaire as a screening tool for anxiety and depressive symptoms in a multi-national study of young adults. *Journal of Affective Disorders, 187*, 197–202. <https://doi.org/10.1016/j.jad.2015.08.045>

Goldberg, D., & Williams, P. (1988). A user’s guide to the General Health Questionnaire. NFER-Nelson.

González, P., Nuñez, A., Merz, E. L., Brintz, C., Weitzman, O., Navas, E. L., Camacho, A., Buelna, C., Penedo, F. J., Wassertheil-Smoller, S., Perreira, K. M., Isasi, C. R., Choca, J. P., Talavera, G. A., & Gallo, L. C. (2017). Measurement properties of the Center for Epidemiologic Studies Depression Scale (CES-D 10): Findings from HCHS/SOL. *Psychological Assessment, 29*(4), 372–381. <https://doi.org/10.1037/pas0000330>

Greenfield, T. K. (2000). Ways of measuring drinking patterns and the difference they make: Experience with graduated frequencies. *Journal of Substance Abuse, 12*(1–2), 33–49. <https://doi.org/10.1016/S0899-3289(00)00039-0>

Hughes, M. E., Waite, L. J., Hawkley, L. C., & Cacioppo, J. T. (2004). A short scale for measuring loneliness in large surveys: Results from two population-based studies. *Research on Aging, 26*(6), 655–672. <https://doi.org/10.1177/0164027504268574>

Idler, E. L., & Benyamini, Y. (1997). Self-rated health and mortality: A review of twenty-seven community studies. *Journal of Health and Social Behavior*, *38(1)*, 21. <https://doi.org/10.2307/2955359>

Jylhä, M. (2009). What is self-rated health and why does it predict mortality? towards a unified conceptual model. *Social Science & Medicine*, *69*(3), 307–316. <https://doi.org/10.1016/j.socscimed.2009.05.013>

Katz, S., Ford, A. B., Moskowitz, R. W., Jackson, B. A., & Jaffe, M. W. (1963). Studies of illness in the aged: The index of ADL—A standardized measure of biological and psychosocial function. *Journal of the American Medical Association, 185*(12), 914–919. <https://doi.org/10.1001/jama.1963.03060120024016>

Kessler, R. C., Andrews, G., Mroczek, D., Üstün, T. B., & Wittchen, H.-U. (1998). The World Health Organization Composite International Diagnostic Interview short-form (CIDI-SF). *International Journal of Methods in Psychiatric Research, 7*(4), 171–185. <https://doi.org/10.1002/mpr.47>

Kessler, R. C., & Üstün, T. B. (2004). The World Mental Health (WMH) Survey Initiative version of the World Health Organization (WHO) Composite International Diagnostic Interview (CIDI). *International Journal of Methods in Psychiatric Research, 13*(2), 93–121. [https://doi.org/10.1002/mpr.168](https://doi.org/10.1002/mpr.168?utm_source=chatgpt.com)

Koivumaa-Honkanen, H., Kaprio, J., Honkanen, R., Viinamäki, H., & Koskenvuo, M. (2004). Life satisfaction and depression in a 15-year follow-up of healthy adults. *Social Psychiatry and Psychiatric Epidemiology, 39*(12), 994–999. <https://doi.org/10.1007/s00127-004-0833-6>

Lawton, M. P., & Brody, E. M. (1969). Assessment of older people: Self-maintaining and instrumental activities of daily living. *The Gerontologist, 9*(3, Part 1), 179–186. <https://doi.org/10.1093/geront/9.3_Part_1.179>

Lorant, V., Deliège, D., Eaton, W., Robert, A., Philippot, P., & Ansseau, M. (2003). Socioeconomic inequalities in depression: A meta-analysis. *American Journal of Epidemiology, 157(2)*, 98–112. <https://doi.org/10.1093/aje/kwf182>

Luger, T. M., Suls, J., & Vander Weg, M. W. **(**2014). How robust is the association between smoking and depression in adults? A meta-analysis using linear, non-linear, and mixed-effects models. *Addictive Behaviors, 39*(10), 1418–1429. <https://doi.org/10.1016/j.addbeh.2014.05.011>

Lubben, J., Blozik, E., Gillmann, G., Lantz, M. S., Stuck, A. E., & Beck, J. C. (2006). Performance of an abbreviated version of the Lubben Social Network Scale among three European community-dwelling populations. *The Gerontologist*, 46(4), 503–513. <https://doi.org/10.1093/geront/46.4.503>

Mohebbi, M., Nguyen, V., McNeil, J. J., Woods, R. L., Nelson, M. R., Shah, R. C., Storey, E., Murray, A. M., Reid, C. M., Kirpach, B. R., Wolfe, R., Lockery, J. E., Berk, M., & ASPREE Investigator Group. (2018). Psychometric properties of a short form of the Center for Epidemiologic Studies Depression (CES-D-10) scale for screening depressive symptoms in healthy community-dwelling older adults. *General Hospital Psychiatry, 51*, 118–125. <https://doi.org/10.1016/j.genhosppsych.2017.08.002>

Mund, M., & Neyer, F. J. **(**2019). Loneliness effects on personality. *International Journal of Behavioral Development, 43*(2), 136–146. <https://doi.org/10.1177/0165025418800224>

Nomaguchi, K., & Milkie, M. A. (2020). Parenthood and well-being: A decade in review. *Journal of Marriage and Family*, 82(1), 198–223. <https://doi.org/10.1111/jomf.12646>

Pavot, W., & Diener, E. (1993). Review of the Satisfaction With Life Scale. *Psychological Assessment, 5*(2), 164–172. <https://doi.org/10.1037/1040-3590.5.2.164>

Prince, M. J., Harwood, R. H., Blizard, R. A., Thomas, A., & Mann, A. H. (1997). Social support deficits, loneliness and life events as risk factors for depression in old age: The Gospel Oak Project VI. *Psychological Medicine, 27*(2), 323–332. <https://doi.org/10.1017/S0033291796004485>

Ridley, M., Rao, G., Schilbach, F., & Patel, V. (2020). Poverty, depression, and anxiety: Causal evidence and mechanisms. *Science*, 370(6522). <https://doi.org/10.1126/science.aay0214>

Romppel, M., Hinz, A., Finck, C., Young, J., Brähler, E., & Glaesmer, H. (2017). Cross-cultural measurement invariance of the General Health Questionnaire-12 in a German and a Colombian population sample. *International Journal of Methods in Psychiatric Research, 26*(4), Article e1532. <https://doi.org/10.1002/mpr.1532>

Schürmann-Vengels, J., Kneuer, A., Cavus, Ö., & Flückiger, C. (2025). The association between positive affect and depressive symptoms in individuals with diagnosed or probable major depressive disorder: A systematic review and meta-analysis. *Behaviour Research and Therapy, 192*, 104789. <https://doi.org/10.1016/j.brat.2025.104789>

Simon, R. W., & Barrett, A. E. (2010). Nonmarital romantic relationships and mental health in early adulthood: Does the association differ for women and men? *Journal of Health and Social Behavior, 51*(2), 168–182. <https://doi.org/10.1177/0022146510372343>

Strohschein, L., McDonough, P., Monette, G., & Shao, Q. (2005). Marital transitions and mental health: Are there gender differences in the short-term effects of marital status change? *Social Science & Medicine, 61*(11), 2293–2303. <https://doi.org/10.1016/j.socscimed.2005.07.020>

Sue, D. W., Sue, D., Sue, D. M., & Sue, S. (2022). Understanding abnormal behavior (11th ed.). Wiley.

Thoits, P. A. (2011). Mechanisms linking social ties and support to mental health. *Journal of Health and Social Behavior, 52*(2), 145–161. <https://doi.org/10.1177/0022146510395592>

Umberson, D., Pudrovska, T., & Reczek, C. (2010). Parenthood, childlessness, and well-being: A life course perspective. *Journal of Marriage and Family, 72*(3), 612–629. <https://doi.org/10.1111/j.1741-3737.2010.00721.x>

Williams, D. R., Lawrence, J. A., & Davis, B. A. (2019). Racism and health: Evidence and needed research. *Annual Review of Public Health, 40*, 105–125. <https://doi.org/10.1146/annurev-publhealth-040218-043750>

Williams, K., & Umberson, D. (2004). Marital status, marital transitions, and health: A gendered life course perspective. *Journal of Health and Social Behavior, 45*(1), 81–98. <https://doi.org/10.1177/002214650404500106>

Wittchen, H.-U., Robins, L. N., Cottler, L. B., Sartorius, N., Burke, J. D., Regier, D., & Participants in the Multicentre WHO/ADAMHA Field Trials. (1991). Cross-cultural feasibility, reliability and sources of variance of the Composite International Diagnostic Interview (CIDI). *The British Journal of Psychiatry, 159*(5), 645–653. <https://doi.org/10.1192/bjp.159.5.645>

Zhu, X., Wang, Y., Luo, Y., Ding, R., Shi, Z., & He, P. (2024). Bidirectional, longitudinal associations between depressive symptoms and IADL/ADL disability in older adults in China: A national cohort study. *BMC Geriatrics*, *24*(1). <https://doi.org/10.1186/s12877-024-05248-y>
